# Influence of TGFB1 and CTLA4 polymorphisms on calcineurin inhibitors dose and risk of acute rejection in renal transplantation

**DOI:** 10.1038/s41598-021-96457-7

**Published:** 2021-09-02

**Authors:** Anna Bogacz, Marlena Wolek, Jerzy Sieńko, Bogusław Czerny, Bogusław Machaliński, Piotr Olbromski, Maciej Kotowski

**Affiliations:** 1 Department of Stem Cells and Regenerative Medicine, Institute of Natural Fibres and Medicinal Plants, Wojska Polskiego 71b, 60-630 Poznan, Poland; 2 Department of Histocompatibility with Laboratory of Genetic Diagnostics, Regional Blood Center, Marcelińska 44, 60-354 Poznan, Poland; 3 Department of General Surgery and Transplantation, Pomeranian Medical University, Powstańców Wlkp. 72, 70-111 Szczecin, Poland; 4 Department of General Pharmacology and Pharmacoeconomics, Pomeranian Medical University, Żołnierska 48, 71-230 Szczecin, Poland; 5 Department of General Pathology, Pomeranian Medical University, Powstańców Wlkp. 72, 70-111 Szczecin, Poland; 6 Clinic of Operational Gynecology, Poznan University of Medical Sciences, Polna 33, 60-535 Poznan, Poland

**Keywords:** Molecular biology, Molecular medicine, Nephrology

## Abstract

Organ transplant is often the treatment of choice as it extends and improves patient life. Immunosuppressive treatment, which prevents acute rejection of the organ, is used in transplant patients to prevent the loss of transplant. The aim of the study was to determine the impact of the CTLA4 (+49A>G, rs231775) and the TGF-β1 (−800G>A, rs1800468) polymorphisms on the therapeutic effect of immunosuppressive drugs (cyclosporine—CsA, tacrolimus—TAC) and the risk of acute rejection in renal transplant patients. The analysis of the CTLA4 +49A>G and the TGF-β1 −800G>A polymorphisms was carried out in 392 patients after kidney transplant using real-time PCR. The CTLA4 +49A>G polymorphism did not affect CsA or TAC dose, ratio of drug concentration to dose (C/D), and blood concentrations. As for the TGF-β1 -800G>A polymorphism, patients with the GA genotype required lower TAC doses compared to the GG genotype (TAC 12 h: 3.63 mg vs 5.3 mg, TAC 24 h: 2.38 mg vs 3.29 mg). Comparing the C/D ratio in both groups (TAC 12 h and TAC 24 h), higher C/D ratio was observed in patients with the GA genotype. These results indicate that patients with the A allele require slightly lower doses of TAC. The results suggest that the TGF-β1 −800 G>A polymorphism may influence the TAC dose, while the +49A>G polymorphism of the CTLA4 gene does not correlate with the dose of CsA or TAC. The analysis of the biochemical parameters of the renal profile showed no impact of the CTLA4 and the TGF-β1 polymorphisms on the risk of organ rejection.

## Introduction

Chronic kidney disease (CKD) is a global public health problem, that influences the renal structure and function, usually leading to end-stage renal disease^1^. Kidney transplant is the targeted method to treat this disease. Optimal immunosuppressive therapy in kidney transplant remains to be determined. The group of immunosuppressive drugs is heterogeneous, and includes medicines such as ciclosporin (CsA) and tacrolimus (TAC)^2^. Cyclosporine is a peptide consisting of 11 amino acids belonging to the group of macrolide antibiotics. Its mechanism of action is associated with the inhibition of the signaling pathway which activates the transcription of the genes involved in the encoding of cytokines, mainly IL-2 in T lymphocytes. Furthermore, CsA stimulates the expression of TGF-β1 (transforming growth factor beta), which contributes to the occurrence of renal fibrosis after transplant^3^. Tacrolimus (TAC) as a calcineurin inhibitor is also a macrolide antibiotic. Although immunosuppressants are necessary to maintain the graft, these drugs can cause numerous side effects^4^. In order to minimize the nephrotoxic effects, it is important to comply with the pharmacokinetics and pharmacogenetics of the drugs, which allows to adjust the dosage to the individual needs of the patient. Among many studies about cytokine gene polymorphisms, the greatest impact on transplant was documented for the polymorphisms of the TGF-β1, TNF-α, IFN-γ, IL-6, IL-10 genes^5,6^. TGF-β1 is a cytokine produced by dendritic cells, NK, and leukocytes. It is the main cytokine involved in the process of fibrosis in various chronic kidney diseases. The transforming growth factor β1 also acts as an immunosuppressive agent, by inhibiting early T-cell proliferation and macrophage activation. This cytokine also intensifies vasoconstriction in the course of interstitial fibrosis and renal tubular atrophy (IF/TA) by stimulating the synthesis of endothelin 1 (ET-1) and inhibiting the production of endogenous nitric oxide. Cyclosporine A increases the production of TGF-β1, which may be associated with nephrotoxicity in the patients and the development of fibrosis in the transplanted organ^6–8^. CTLA4 is another potential gene which affects transplant results. CTLA4 (CD152) is an immunoglobulin-like molecule. The +49A>G polymorphism in exon 1, in which alanine is replaced by threonine amino acid, results in the expression of a defective receptor. This affects the inhibition of T-cell activation. It has been documented that individuals with the GG genotype have lymphocytes with significantly increase proliferation under the influence of specific factors as compared to lymphocytes of individuals with the AA genotype^9–11^. The aim of the study was to determine the impact of the CTLA4 (+49A>G, rs231775) and the TGF-β1 (−800G>A, rs1800468) polymorphisms on the therapeutic effect of immunosuppressive drugs (cyclosporine CsA, tacrolimus TAC) and the risk of acute rejection in renal transplant patients.

## Methods

The analysis was performed in a group of 392 patients (163 women and 229 men, aged: 19–82 years) after kidney transplant. The patients were recruited at the Division of Nephrology and Kidney Transplantation, Independent Public Provincial Hospital in Szczecin, and the Department of General Surgery and Transplantation, Pomeranian Medical University in Szczecin. The research was approved by the Bioethics Committee of Poznan University of Medical Sciences, Poland (no. 510/12; 574/18.), and all patients gave written informed consent to participate before enrolling in the study. The standard regimen of immunosuppression following kidney transplantation was the use of a calcineurin inhibitor (tacrolimus/CsA) in combination with mycophenolate mofetil and glucocorticoids. Blood samples were collected between the third and sixth months after transplantation. Peripheral blood was used for biochemical tests (renal, hepatic and lipid profile parameters), measurement of drug blood concentrations (cyclosporine, tacrolimus), and the analysis of the genetic variants. Fasting whole blood concentration of TAC and CsA was determined before drug administration. The analysis was performed using the ARCHITECT i2000SR analyzer (Abbott). The ARCHITECT System Tacrolimus was used to determine drug concentrations based on chemiluminescent microparticle immunoassay (CMIA), according to the manufacturer's protocol.

Acute kidney transplant rejection occurs most frequently in the first weeks after transplantation and can be divided into T cell-mediated rejection (TCMR) and antibody-mediated rejection (ABMR). TCMR is characterized by lymphocytic infiltration of the tubules, interstitium, and sometimes the intima of the artery. The ABMR usually shows evidence of circulating donor-specific alloantibodies and immunological evidence of antibody mediated kidney damage. According to the KDIGO (Kidney Disease Improving Global Outcomes) (https://kdigo.org/) guidelines, acute kidney injury is associated with an increase in serum creatinine > 0.3 mg/dL within 48 h, and a percentage increase in creatinine concentration in serum ≥ 50% (1.5-fold from the baseline value) or decreased urine output (oliguria < 0.5 mL/kg/h for more than 6 h). Moreover, it is recommended to quantify protein excretion every 3 months during the first year^12^. Hence, the risk criteria for rejection of a transplanted kidney include, among others, clinical symptoms such as: proteinuria, hypertension, decreased glomerular filtration, and increased creatinine levels. Clinical and biochemical parameters were evaluated in order to determine the risk for graft rejection. Genetic analyses were carried out at the Department of Stem Cells and Regenerative Medicine, the Institute of Natural Fibers in Poznan, Poland. Genomic DNA was isolated from peripheral blood leukocytes using a commercial kit Macherey–Nagel NucleoSpin^®^Blood (Macherey–Nagel GmbH&Co, Germany) according to the manufacturer's protocol. DNA concentration was measured using a DeNovix DS-11 Spectrophotometer (DeNovix Inc., Wilmington, USA). The analysis of the CTLA4 +49A>G and the TGF-β1 −800G>A polymorphisms was performed by real-time PCR using LightCycler^®^96 (Roche Diagnostics). A set of LightSNiP rs231775 for CTLA4 polymorphism and a set LightSNiP rs1800468 for TGF-β1 polymorphism contained appropriate concentrations of specific primers and probes for the amplified fragment and were prepared according to the manufacturer's instructions. The PCR program was initiated at 95 °C for 10 min. Each PCR cycle comprised a denaturation step at 95 °C for 10 s, an annealing step at 60 °C for 10 s and an elongation step at 72 °C for 15 s (45 cycles). The final stage was the melting of products as a result of temperature rise to 95 °C. The reaction composition of a single sample was as follows: H_2_O—6.7 µl, LightSNiP CTLA4 rs231775 or TGF-β1 rs1800468—0.5 µl, LightCycler480 Genotyping Master—1 µl, MgCl2 (25 mM)—0.8 µl, DNA (50 ng)—1 µl^34^. The analysis of the genotyping was based on the melting curve using LightCycler^®^96 Basic Software. Statistical analysis of the results was performed using the SPSS 17.0 PL program. The expected frequency of the genotypes was calculated using the Hardy–Weinberg equation, which was compared with the values observed using the Pearson χ^2^ test. The expected results were presented with a 95% confidence interval (95% CI). The correlations between the studied polymorphisms and the clinical parameters were also analyzed, and presented by mean values, standard deviation using one-way ANOVA analysis. The p value of < 0.05 was considered as statistically significant^13^.

All methods were carried out in accordance with relevant guidelines and regulations.

## Results

**Figure 1:**
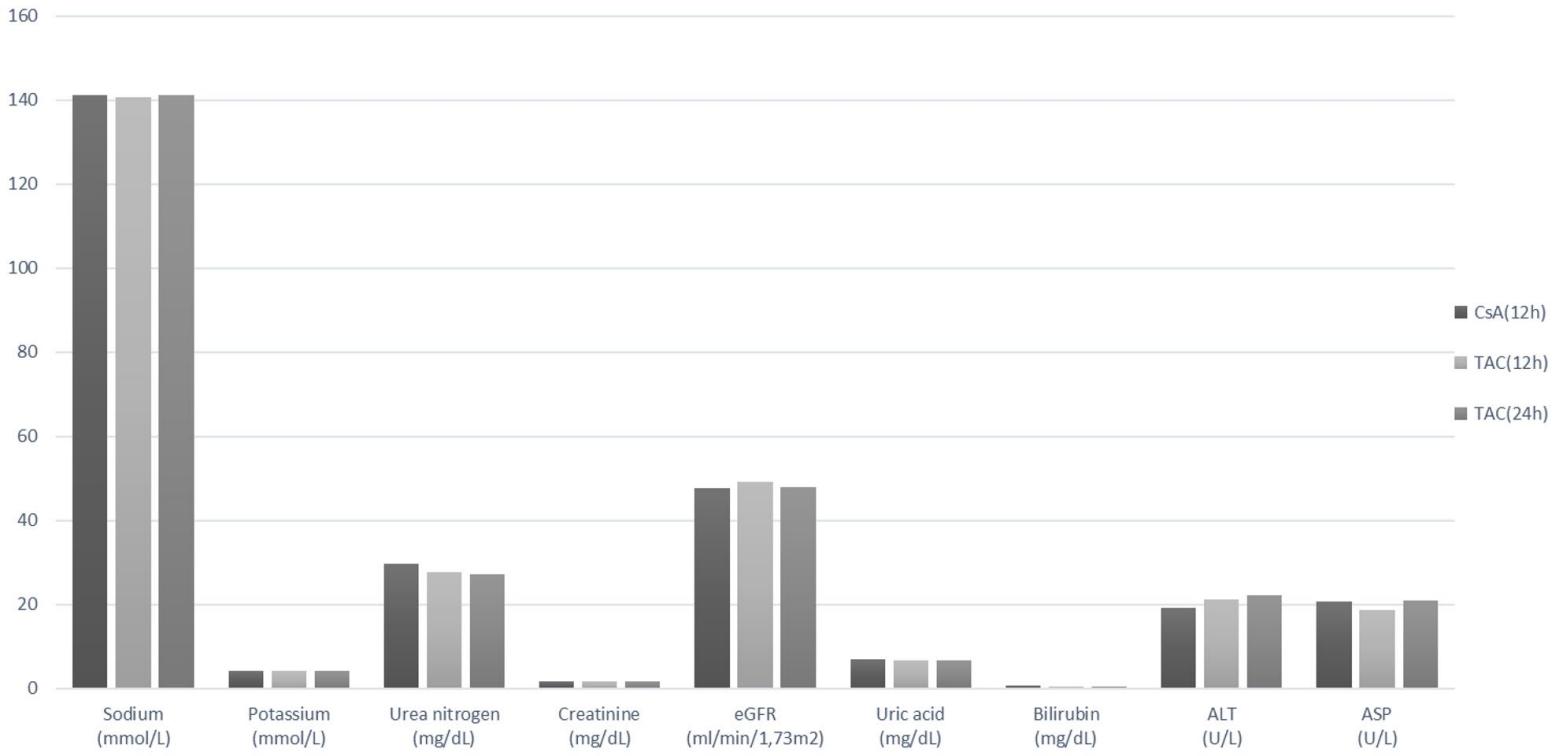
Characteristics of selected clinical and biochemical parameters in renal transplant patients receiving cyclosporine A (CsA) or tacrolimus (TAC) every 12 or 24 h. (CsA-12 h, n = 183), (TAC-12 h, n = 53), (TAC-24 h, n = 156). *ALT* alanine transaminase, *AST* aspartate transaminase, *eGFR* estimated glomerular filtration rate.

The post-transplant follow-up period was between the third and sixth month after kidney transplantation. The analysis of clinical and biochemical parameters showed no significant differences between TAC and CsA patients (Table 1). Parameters such as BMI, weight, and age, which could affect the dosage and drug metabolism, were comparable in the analyzed groups. The patients were divided into three groups: receiving TAC every 12 (TAC 12) and every 24 (TAC 24) hours, or CsA every 12 h (CsA 12). The patients receiving TAC every 24 h were taking the extended- release formulation. The analysis of the ANOVA variance showed comparable mean values for hepatic, renal and lipid profile parameters, and large differences at their maximum concentrations, especially for TAC 24 patients. The highest values were observed for the following parameters: ALT (TAC 24 h: 206 U/L vs CsA 12 h: 73 U/L, TAC 12 h: 98 U/L), ASP (TAC 24 h: 139 U/L vs CsA 12 h: 70 U/L, TAC 12 h: 55 U/L), total cholesterol (TAC 24 h: 409 mg/dL vs CsA 12 h: 344 mg/dL, TAC 12 h: 287 mg/dL), triglycerides (TAC 24 h: 839 mg/dL vs CsA 12 h: 404 mg/dL, TAC 12 h: 327 mg/dL), and total lipids (TAC 24 h: 1511 mg/dL vs CsA 12 h: 1183 mg/dL, TAC 12 h: 995 mg/dL). Additionally, bilirubin was significantly elevated in CsA than TAC groups (CsA: 0.60 ± 0.28 mg/dL vs TAC 12 h: 0.50 ± 0.29 mg/dL, TAC 24 h: 0.51 ± 0.24 mg/dL). The analysis of renal parameters such as (sodium, potassium, urea nitrogen, creatinine, uric acid, eGFR) showed no differences between the studied groups (Fig. 1). Assessment of the correlation between taking the immunosuppressant drug and CTLA4 +49A>G polymorphism did not show statistically significant differences in the distribution of the individual genotypes (Table 2). The AG genotype was the most common in each of the analyzed groups: CsA 12 h: 49.2%, TAC 12 h: 49.1% and TAC 24 h: 50%, p > 0.05. The frequency distribution of genotypes +49A>G gene CTLA4 was consistent with the law of Hardy–Weinberg equilibrium. Analysis of the genotype frequency of the −800G>A (rs1800468) polymorphism for the TGF-β1 gene did not show statistically significant differences between the study groups (Table 2). The highest frequency of the GG genotype was observed in all groups (CsA 12 h: 90.7%, TAC 12 h: 84.9%, TAC 24 h 88.5%, p > 0.05). In addition, the AA genotype was not found in patients taking tacrolimus. The analysis of mean CsA blood concentration, dose, and concentration/dose (C/D) ratio with the genetic variants did not show statistically significant differences, indicating that the CTLA4 +49A>G polymorphism does not affect the CsA dose (Table 3). It was noticed that TAC 12 patients with the AA genotype had the highest mean TAC blood concentration (AA: 7.27 ng/mL vs GG: 5.48 ng/mL, p > 0.05) and C/D ratio (AA: 1.95 vs AG: 1.74, p > 0.05) as compared to other genotypes of the CTLA4 +49A>G polymorphism (Table 3). In addition, carriers with the AA and AG genotypes required higher doses of TAC as compared to the GG genotype (AA: 4.83 mg, AG: 5.59 mg vs 3.88 mg, p > 0.05). No relationship between dosage and individual genotypes of the CTLA4 +49A>G polymorphism was found in TAC 24 patients (Table 3). Analysis of the data on TAC dose dependence and genetic variants of the TGF-β1 −800G>A polymorphism revealed that patients with the GA genotype required lower doses of the drug as compared to the GG genotype (TAC 12 h: 3.63 mg vs 5.3 mg, TAC 24 h: 2.38 mg vs 3.29 mg. Comparing the C/D ratio, we showed that in both groups the C/D ratio was higher for patients with the GA genotype (Table 4). Moreover, analysis of the clinical parameters (BMI, systolic and diastolic pressure) and biochemical parameters of the renal profile (potassium, uric, acid creatinine, eGFR) and the CTLA4 and TGF-β1 polymorphisms showed no relation to the risk of organ rejection and indicated a favorable prognosis of the transplanted kidney (Tables 5, 6). It should be emphasized that BMI may affect the risk of new onset diabetes after transplantation, treatment outcomes, including patient and transplant survival, delay in transplant operation, and transplant rejection. According to European Renal Best Practice (ERBP) recommendations, patients with a pre-transplant body mass index (BMI) greater than 30 kg/m^2^ should reduce their body weight. In addition, proteinuria, which falls under the risk of transplant rejection criteria, has not been observed in our patients.

**Table 1:** Clinical and biochemical parameters in renal transplant patients receiving cyclosporine A (CsA) or tacrolimus (TAC) every 12 or 24 h.

Parameter	Drug	Mean ± SD	95% CI	Minimum	Maximum	*P*
Age (years)	CsA (12 h)	53.69 ± 12.74	51.84–55.53	22	82	
	TAC (12 h)	47.10 ± 14.94	43.10–51.10	19	81	
	TAC (24 h)	52.35 ± 12.81	50.40–54.30	19	81	
	Total	52.24 ± 13.23	50.96–53.53	19	82	
Weight (kg)	CsA (12 h)	78.84 ± 17.16	76.35–81.33	40	139	0.083
	*TAC* = *12*	75.61 ± 15.77	71.38–79.83	44	132	
	TAC (24 h)	75.13 ± 15.23	72.81–77.46	47	135	
	Total	76.88 ± 16.27	75.30–78.46	40	139	
BMI (kg/m^2^)	CsA (12 h)	27.20 ± 5.11	26.46–27.94	17.31	42.9	0.075
	TAC (12 h)	25.72 ± 4.31	24.57–26.88	17.63	40.74	
	TAC (24 h)	26.38 ± 4.41	25.71–27.06	18.59	43.58	
	Total	26.66 ± 4.75	26.20–27.13	17.31	43.58	
Sodium (mmol/L)	CsA (12 h)	141.10 ± 3.54	140.59–141.62	120	149	0.078
	TAC (12 h)	140.71 ± 3.39	139.80–141.62	129	147	
	TAC (24 h)	141.10 ± 2.89	140.66–141.55	132	149	
	Total	141.05 ± 3.26	140.73–141.37	120	149	
Potassium (mmol/L)	CsA (12 h)	4.16 ± 0.44	4.10–4.23	3.14	5.42	0.451
	TAC (12 h)	4.20 ± 0.60	4.04–4.36	3.31	5.71	
	TAC (24 h)	4.11 ± 0.47	4.04–4.19	2.93	6.09	
	Total	4.15 ± 0.48	4.10–4.19	2.93	6.09	
Bilirubin (mg/dL)	CsA (12 h)	0.60 ± 0.28	0.56–0.64	0.16	1.69	0.002
	TAC (12 h)	0.50 ± 0.29	0.43–0.58	0.15	1.51	
	TAC (24 h)	0.51 ± 0.24	0.47–0.54	0.15	1.58	
	Total	0.55 ± 0.27	0.52–0.58	0.15	1.69	
Urea nitrogen (mg/dL)	CsA (12 h)	29.82 ± 12.95	27.94–31.70	8.3	71.1	0.201
	TAC (12 h)	27.74 ± 16.36	23.36–32.13	11.9	108.6	
	TAC (24 h)	27.18 ± 14.60	24.95–29.41	7.5	111.9	
	Total	28.46 ± 14.16	27.08–29.83	7.5	111.9	
Creatinine (mg/dL)	CsA (12 h)	1.60 ± 0.62	1.51–1.69	0.67	4.31	0.942
	TAC (12 h)	1.62 ± 0.68	1.44–1.80	0.73	3.52	
	TAC (24 h)	1.59 ± 0.62	1.49–1.68	0.71	3.97	
	Total	1.60 ± 0.63	1.54–1.66	0.67	4.31	
eGFR	CsA (12 h)	47.79 ± 15.18	45.6–49.99	14.22	75	0.863
	TAC (12 h)	49.09 ± 16.05	44.79–53.39	15.05	74	
	TAC (24 h)	48.06 ± 16.12	45.59–50.52	14.4	75	
	Total	48.08 ± 15.65	46.55–49.60	14.22	75	
Uric acid (mg/dL)	CsA (12 h)	6.95 ± 1.49	6.73–7.16	3.7	10.9	0.453
	TAC (12 h)	6.77 ± 1.60	6.34–7.20	4	10.8	
	TAC (24 h)	6.76 ± 1.50	6.53–6.99	3.2	13.4	
	Total	6.84 ± 1.51	6.70–6.99	3.2	13.4	
ALT (U/L)	CsA (12 h)	19.25 ± 10.94	17.67–20.84	3	73	0.258
	TAC (12 h)	21.14 ± 14.77	17.18–25.09	8	98	
	TAC (24 h)	22.20 ± 22.23	18.82–25.59	5	206	
	Total	20.72 ± 16.95	19.07–22.37	3	206	
ASP (U/L)	CsA (12 h)	20.68 ± 8.73	19.41–21.94	10	70	0.393
	TAC (12 h)	18.82 ± 7.37	16.84–20.79	9	55	
	TAC (24 h)	21.07 ± 13.36	19.03–23.10	9	139	
	Total	20.58 ± 10.73	19.54–21.62	9	139	
Total cholesterol (mg/dL)	CsA (12 h)	187.96 ± 39.37	182.15–193.77	80	344	0.437
	TAC (12 h)	183.11 ± 34.84	173.60–192.62	118	287	
	TAC (24 h)	190.89 ± 40.80	184.65–197.12	119	409	
	Total	188.53 ± 39.39	184.65–192.40	80	409	
Cholesterol-HDL (mg/dL)	CsA (12 h)	62.94 ± 20.48	59.29–66.58	27.6	118.8	0.265
	TAC (12 h)	57.01 ± 18.05	50.71–63.31	29	95	
	TAC (24 h)	61.62 ± 16.82	58.49–64.76	20.6	104.4	
	Total	61.64 ± 18.75	59.40–63.89	20.6	118.8	
Cholesterol-LDL (mg/dL)	CsA (12 h)	95.66 ± 35.84	89.26–102.06	36	239	0.232
	TAC (12 h)	96.35 ± 33.82	84.55–108.15	37	191	
	TAC (24 h)	98.5 ± 31.39	92.62–104.37	27	188	
	Total	96.93 ± 33.70	92.88–100.97	27	239	
Triglycerides (mg/dL)	CsA (12 h)	142.37 ± 65.41	130.75–154.00	48	404	0.545
	TAC (12 h)	152.61 ± 70.04	128.17–177.05	53	327	
	TAC (24 h)	152.93 ± 91.75	135.83–170.04	55	839	
	Total	148.06 ± 77.90	138.74–157.38	48	839	
Total lipids (mg/dL)	CsA (12 h)	638.50 ± 132.78	614.90–662.11	430	1183	0.598
	TAC (12 h)	641.35 ± 129.49	596.17–686.53	393	995	
	TAC (24 h)	656.18 ± 143.83	629.37–682.99	409	1511	
	Total	646.23 ± 136.88	629.86–662.60	393	1511	

The post-transplant follow-up period was between the third and sixth month after kidney transplantation.

(CsA-12 h, n = 183), (TAC-12 h, n = 53), (TAC-24 h, n = 156).

Values normally distributed are expressed as means ± standard deviation (SD). P-significance level for the difference between the groups (one-way ANOVA).

*BMI* body mass index, *ALT* alanine transaminase, *AST* aspartate transaminase, *eGFR* estimated glomerular filtration rate.

**Table 2:** Genotype frequencies of CTLA4 +49A>G (rs231775) and TGF-β1 −800G>A (rs1800468) polymorphisms in renal transplant patients receiving cyclosporine A (CsA) or tacrolimus (TAC) every 12 or 24 h.

Genotype	Patients receiving CsA (12 h)		Patients receiving TAC (12 h)		Patients receiving TAC (24 h)		*P*
	Observed values n (%)	Expected values %	Observed values n (%)	Expected values %	Observed values n (%)	Expected values %	
**+49A>G CTLA4**							
AA	59 (32.2)	32.49	18 (34)	34.81	48 (30.8)	31.36	
AG	90 (49.2)	49.02	26 (49.1)	48.38	78 (50)	49.28	0.426
GG	34 (18.6)	18.49	9 (17)	16.81	30 (19.2)	19.36	
Total	183 (100)	100	53 (100)	100	156 (100)	100	
**−800G>A TGF-β1**							
GG	166 (90.7)	90.25	45 (84.9)	84.64	138 (88.5)	88.36	
GA	15 (8.2)	9.5	8 (15.1)	14.72	18 (11.5)	11.28	0.388
AA	2 (1.1)	0.25	0 (0)	0.64	0 (0)	0.36	
Total	183 (100)	100	53 (100)	100	156 (100)	100

**Table 3:** Comparison of tacrolimus (TAC) and cyclosporine A (CsA) concentration in blood and its dosing between genotypes of CTLA4 +49A>G polymorphism (rs231775) in patients receiving the drug every 12 and 24 h (CsA-12 h, n = 183), (TAC-12 h, n = 53), (TAC-24 h, n = 156).

Parameter	Genotype	Mean ± SD	95% CI	Min	Max	*P*
**+49A>G CTLA4 (CsA = 12 h)**						
CsA blood concentration (ng/mL)	AA	114.81 ± 50.95	101.53–128.09	58.8	262.9	0.962
	AG	114.11 ± 48.34	103.99–124.24	60.7	315.9	
	GG	116.84 ± 46.16	100.73–132.95	53.2	215.5	
CsA dose (mg/day)	AA	162.62 ± 64.94	145.70–179.55	50	500	0.386
	AG	176.66 ± 60.40	164.01–189.31	50	400	
	GG	169.85 ± 53.92	151.03–188.66	100	400	
Concentration/dose of CsA (C/D)	AA	0.74 ± 0.32	0.66–0.83	0.02	1.75	0.320
	AG	0.67 ± 0.25	0.62–0.72	0.01	1.57	
	GG	0.71 ± 0.27	0.61–0.80	0.34	1.4	
**+49A>G CTLA4 (TAC = 12 h)**						
TAC blood concentration (ng/mL)	AA	7.27 ± 2.46	6.05–8.50	3.1	11.3	0.394
	AG	6.69 ± 3.87	5.13–8.26	2.1	22.4	
	GG	5.48 ± 1.83	4.07–6.90	2.9	8	
TAC dose (mg/day)	AA	4.83 ± 3.18	3.24–6.41	2	14	0.548
	AG	5.59 ± 4.84	3.63–7.55	1.5	24	
	GG	3.88 ± 3.37	1.29–6.47	1	12	
Concentration/dose of TAC (C/D)	AA	1.95 ± 1.16	1.37–2.53	0.57	4.9	0.778
	AG	1.74 ± 1.03	1.32–2.16	0.25	3.73	
	GG	1.93 ± 0.82	1.30–2.56	0.51	2.9	
**+49A>G CTLA4 (TAC = 24 h)**						
TAC blood concentration (ng/mL)	AA	5.63 ± 2.60	4.88–6.39	2	16.6	0.527
	AG	5.50 ± 2.17	5.01–5.99	2.1	15.1	
	GG	6.08 ± 2.53	5.13–7.02	2.1	11.5	
TAC dose (mg/day)	AA	3.30 ± 1.72	2.80–3.80	1	8	0.605
	AG	3.04 ± 2.03	2.58–3.50	1	12	
	GG	3.4 ± 1.69	2.76–4.03	1	8	
Concentration/dose of TAC (C/D)	AA	2.11 ± 1.35	1.72–2.51	0.58	6.4	0.201
	AG	2.58 ± 1.98	2.13–3.03	0.54	9.5	
	GG	2.07 ± 1.14	1.64–2.50	0.55	5.6

**Table 4:** Comparison of tacrolimus (TAC) and cyclosporine A (CsA) concentration in blood and its dosing between genotypes of TGF-β1 -800G>A polymorphism (rs1800468) in patients receiving the drug every 12 and 24 h (CsA-12 h, n = 183), (TAC-12 h, n = 53), (TAC-24 h, n = 156).

Parameter	Genotype	Mean ± SD	95% CI	Min	Max	*P*
**−800G>A TGF-β1 (CsA = 12 h)**						
CsA blood concentration (ng/mL)	GG	113.92 ± 48.55	106.48–121.36	50	315.9	0.716
	GA	123.19 ± 51.36	94.74–151.63	65.7	262.9	
	AA	129.05 ± 39.24	–	101.3	156.8	
CsA dose (mg/day)	GG	170.15 ± 61.31	160.75–179.54	50	500	0.375
	GA	185 ± 55.74	154.13–215.86	100	300	
	AA	125 ± 35.35	–	100	150	
Concentration/dose of CsA (C/D)	GG	0.69 ± 0.27	0.65–0.74	0.01	1.6	0.115
	GA	0.70 ± 0.33	0.51–0.88	0.33	1.75	
	AA	1.12 ± 0.63	–	0.68	1.57	
**−800G>A TGF-β1 (TAC = 12 h)**						
TAC blood concentration (ng/mL)	GG	6.51 ± 2.34	5.81–7.22	2.1	11.3	0.358
	GA	7.65 ± 6.25	2.42–12.87	3	22.4	
TAC dose (mg/day)	GG	5.3 ± 4.35	3.99–6.60	1	24	0.291
	GA	3.62 ± 1.68	2.21–5.03	2	6	
Concentration/dose of TAC (C/D)	GG	1.81 ± 1.07	1.49–2.13	0.3	4.9	0.59
	GA	2.03 ± 0.83	1.33–2.73	1.1	3.7	
**−800G>A TGF-β1 (TAC = 24 h)**						
TAC blood concentration (ng/mL)	GG	5.73 ± 2.45	5.32–6.14	2.0	16.6	0.255
	GA	5.05 ± 1.56	4.27–5.83	2	8.8	
TAC dose (mg/day)	GG	3.29 ± 1.93	2.97–3.62	1	12	0.053
	GA	2.38 ± 1.07	1.85–2.92	1	5	
Concentration/dose of TAC (C/D)	GG	2.32 ± 1.73	2.03–2.61	0.5	9.5	0.721
	GA	2.47 ± 1.26	1.84–3.10	1.0	5.9

**Table 5:** Analysis of the selected clinical par−ameters and biochemical parameters of the renal profile in relation to the genotypes of CTLA4 +49A>G polymorphism (rs231775).

Parameter	Genotype	Mean ± SD	95% CI	*P*
**+9A>G CTLA4 (CsA = 12 h)**				
Potassium (mmol/L)	AA	4.10 ± 0.46	3.98–4.23	0.365
	AG	4.21 ± 0.45	4.11–4.30	
	GG	4.18 ± 0.36	4.05–4.31	
Creatinine (mg/dL)	AA	1.67 ± 0.71	1.48–1.86	0.597
	AG	1.56 ± 0.53	1.45–1.68	
	GG	1.60 ± 0.70	1.36–1.85	
Uric acid (mg/dL)	AA	7.06 ± 1.66	6.62–7.50	0.291
	AG	6.79 ± 1.36	6.51–7.08	
	GG	7.23 ± 1.47	6.71–7.75	
eGFR (mL/min/1.73 m^2^)	AA	46.19 ± 14.77	42.34–50.04	0.637
	AG	48.57 ± 15.12	45.40–51.73	
	GG	48.22 ± 16.51	42.46–53.99	
SBP (mmHg)	AA	133.47 ± 14.14	129.78–137.16	0.209
	AG	133.61 ± 13.00	130.88–136.33	
	GG	129.11 ± 11.57	125.07–133.15	
DBP (mmHg)	AA	84.91 ± 9.44	82.45–87.37	0.027
	AG	82.33 ± 7.57	80.74–83.92	
	GG	80.29 ± 7.58	77.64–82.93	
BMI (kg/m^2^)	AA	28.02 ± 5.59	26.56–29.48	0.349
	AG	26.78 ± 4.92	25.75–27.81	
	GG	27.12 ± 4.64	25.50–28.75	
**+49A>G CTLA4 (TAC = 12 h)**				
Potassium (mmol/L)	AA	4.03 ± 0.41	3.82–4.23	0.179
	AG	4.37 ± 0.71	4.08–4.66	
	GG	4.14 ± 0.55	3.71–4.56	
Creatinine (mg/dL)	AA	1.66 ± 0.72	1.30–2.02	0.317
	AG	1.62 ± 0.66	1.36–1.89	
	GG	1.28 ± 0.32	1.03–1.53	
Uric acid (mg/dL)	AA	6.71 ± 1.56	5.93–7.49	0.879
	AG	6.64 ± 1.39	6.07–7.20	
	GG	6.96 ± 2.38	5.13–8.79	
eGFR (mL/min/1.73 m^2^)	AA	48.60 ± 16.09	40.60–56.60	0.112
	AG	47.33 ± 16.29	40.74–53.91	
	GG	59.79 ± 10.09	52.04–67.55	
SBP (mmHg)	AA	127.5 ± 11.53	121.76–133.23	0.183
	AG	133.07 ± 11.32	128.50–137.64	
	GG	126.66 ± 11.45	117.86–135.47	
DBP (mmHg)	AA	81.94 ± 7.69	78.11–85.77	0.901
	AG	82.5 ± 8.74	78.96–86.03	
	GG	81.11 ± 6.00	76.49–85.73	
BMI (kg/m^2^)	AA	26.12 ± 4.37	23.95–28.29	0.934
	AG	25.82 ± 4.72	23.91–27.73	
	GG	25.45 ± 3.86	22.48–28.43	
**+49A>G CTLA4 (TAC = 24 h)**				
Potassium (mmol/L)	AA	4.30 ± 0.56	4.14–4.47	0.002
	AG	4.00 ± 0.41	3.91–4.10	
	GG	4.07 ± 0.33	3.94–4.19	
Creatinine (mg/dL)	AA	1.64 ± 0.56	1.48–1.80	0.25
	AG	1.63 ± 0.69	1.48–1.79	
	GG	1.43 ± 0.50	1.24–1.61	
Uric acid (mg/dL)	AA	6.97 ± 1.77	6.44–7.49	0.567
	AG	6.74 ± 1.33	6.44–7.04	
	GG	6.63 ± 1.20	6.17–7.08	
eGFR (mL/min/1.73 m^2^)	AA	47.20 ± 14.58	42.92–51.48	0.485
	AG	46.98 ± 17.00	43.15–50.82	
	GG	50.96 ± 14.92	45.39–56.54	
SBP (mmHg)	AA	134.37 ± 13.47	130.46–138.28	0.527
	AG	132.01 ± 9.87	129.77–134.25	
	GG	132.83 ± 11.11	128.68–136.98	
DBP (mmHg)	AA	82.60 ± 6.60	80.68–84.52	0.652
	AG	83.11 ± 7.30	81.45–84.77	
	GG	81.66 ± 8.33	78.55–84.78	
BMI (kg/m^2^)	AA	26.16 ± 3.79	25.06–27.26	0.673
	AG	26.57 ± 4.42	25.58–27.57	
	GG	27.08 ± 5.41	25.06–29.10	

(CsA-12 h, n = 183), (TAC-12 h, n = 53), (TAC-24 h, n = 156).

Values normally distributed are expressed as means ± standard deviation (SD). P-significance level for the difference between the groups (one-way ANOVA).

*BMI* body mass index, *eGFR* estimated glomerular filtration rate, *SBP* systolic blood pressure, *DBP* diastolic blood pressure.

**Table 6:** Analysis of the selected clinical parameters and biochemical parameters of the renal profile in relation to the genotypes of TGF-β1 -800G>A polymorphism (rs1800468).n

Parameter	Genotype	Mean ± SD	95% CI	*P*
**−800G>A TGF-β (CsA = 12 h)**				
Potassium (mmol/L)	GG	4.15 ± 0.43	4.08–4.22	
	GA	4.38 ± 0.48	4.11–4.65	
	AA	4.19 ± 0.07	3.55–4.82	
Creatinine (mg/dL)	GG	1.58 ± 0.63	1.48–1.68	0.247
	GA	1.87 ± 0.54	1.57–2.17	
	AA	1.61 ± 0.50	–	
Uric acid (mg/dL)	GG	6.95 ± 1.50	6.72–7.19	0.498
	GA	7.16 ± 1.24	6.47–7.85	
	AA	5.85 ± 2.47	–	
eGFR (mL/min/1.73 m^2^)	GG	48.40 ± 15.30	46.05–50.74	0.13
	GA	40.14 ± 12.54	33.19–47.09	
	AA	49.92 ± 21.31	–	
SBP (mmHg)	GG	132.53 ± 13.07	130.52–134.53	0.772
	GA	134.33 ± 15.22	125.90–142.76	
	AA	137.5 ± 10.60	–	
DBP (mmHg)	GG	82.83 ± 8.22	81.57–84.09	0.871
	GA	82 ± 10.14	76.38–87.61	
	AA	85 ± 7.07	21.46–148.53	
BMI (kg/m^2^)	GG	27.13 ± 4.96	26.37–27.90	0.632
	GA	28.17 ± 6.67	24.48–31.87	
	AA	29.39 ± 5.29	–	
**−800G>A TGF-β (TAC = 12 h)**				
Potassium (mmol/L)	GG	4.20 ± 0.61	4.02–4.38	0.734
	GA	4.28 ± 0.64	3.74–4.82	
Creatinine (mg/dL)	GG	1.61 ± 0.65	1.41–1.80	0.442
	GA	1.41 ± 0.58	0.92–1.90	
Uric acid (mg/dL)	GG	6.76 ± 1.70	6.25–7.27	0.646
	GA	6.47 ± 1.07	5.57–7.37	
eGFR (mL/min/1.73 m^2^)	GG	48.80 ± 15.34	44.19–53.41	0.244
	GA	55.91 ± 17.84	40.99–70.83	
SBP (mmHg)	GG	131.11 ± 11.37	127.69–134.52	0.131
	GA	124.37 ± 11.78	114.52–134.22	
DBP (mmHg)	GG	83 ± 7.86	80.63–85.36	0.041
	GA	76.87 ± 5.93	71.91–81.83	
BMI (kg/m^2^)	GG	26.11 ± 4.43	24.78–27.44	0.324
	GA	24.43 ± 4.13	20.97–27.89	
**−800G>A TGF-β (TAC = 24 h)**				
Potassium (mmol/L)	GG	4.12 ± 0.48	4.04–4.20	0.563
	GA	4.05 ± 0.36	3.87–4.23	
Creatinine (mg/dL)	GG	1.56 ± 0.56	1.47–1.66	0.068
	GA	1.85 ± 0.91	1.39–2.30	
Uric acid (mg/dL)	GG	6.78 ± 1.50	6.53–7.03	0.847
	GA	6.85 ± 1.07	6.32–7.38	
eGFR (mL/min/1.73 m^2^)	GG	48.25 ± 15.22	45.68–50.82	0.356
	GA	44.56 ± 20.47	34.38–54.74	
SBP (mmHg)	GG	132.99 ± 11.54	131.04–134.94	0.787
	GA	132.22 ± 9.58	127.45–136.98	
DBP (mmHg)	GG	82.66 ± 7.30	81.43–83.89	0.951
	GA	82.77 ± 7.32	79.13–86.41	
BMI (kg/m^2^)	GG	26.31 ± 4.10	25.61–27.00	0.064
	GA	28.37 ± 6.30	25.23–31.50	

(CsA-12 h, n = 183), (TAC-12 h, n = 53), (TAC-24 h, n = 156).

*BMI* body mass index, *eGFR* estimated glomerular filtration rate, *SBP* systolic blood pressure, *DBP* diastolic blood pressure.

Values normally distributed are expressed as means ± standard deviation (SD). P-significance level for the difference between the groups (one-way ANOVA).

## Discussion

T-lymphocyte-related cytotoxic 4 (CTLA4) is a key component of the immune system which induces immune tolerance and is one of the critical negative factors regulating the T-cell-mediated immune response. The CTLA4 +49A>G (rs231775) and +6230G>A (rs3087243) polymorphisms play a significant role in transplant rejection and affect the long-term clinical outcome of organ transplant. The +49A>G (rs231775) polymorphism has been the most extensively studied in kidney transplant patients. Numerous studies showed a relationship between the CTLA4 +49A>G polymorphism and acute rejection (AR), which is associated with reduced graft survival in the first months after transplantation. Duan et al. and Zhu et al. performed a meta-analysis to assess the risk of AR after kidney and liver transplant^14,15^. In the study by Duan et al.^14^, the relationship between the CTLA4 +49G allele and AR was weakly significant. In contrast, Zhu et al.^15^, found no significant correlation between the +49G>A polymorphism and AR in kidney transplant, which is consistent with the findings of our study. We also showed no correlation between the +49G>A polymorphism and AR in kidney transplant. Moreover, the analysis of the CTLA4 +49A>G polymorphism did not affect the dose of CsA or TAC administered to the Polish renal transplant patients.

Late acute rejection (LAR) is associated with reduced graft survival and occurs between 1 and 2 years after transplantation as a result of insufficient immunosuppression. In study conducted by Kim et al., it was shown that the CTLA4 gene polymorphism was statistically associated with LAR in Korean patients. The presence of the G allele increased the risk of LAR after organ transplant^16^. Furthermore, a meta-analysis of kidney transplant patients demonstrated that recipients with the GG genotype and the G allele had an increased risk of AR^17^. Kusztal et al., found that the rs231775 polymorphism of the CTLA4 gene was also associated with long-term renal allograft function in Caucasian patients^18^. Yet another study demonstrated the effects of five CTLA4 polymorphisms on long-term renal function after transplant in the Chinese population. These authors observed that 60 months after kidney transplant, eGFR was higher in patients with the rs733618C, rs3087243A and rs5742909TT alleles, and lower in patients with the rs733618TT, rs3087243GG and rs231775GG genotypes^19^. Moreover, cell surface expression of CTLA4 was shown to be significantly increased in individuals with the AA genotype as compared to AG and GG genotypes. T lymphocytes with the +49GG genotype were demonstrated to have higher activation and proliferation than T-cells with the +49AA variant. The CTLA4 +49G>A polymorphism causes substitution of 17Thr > 17Ala. Recombinant CTLA4 containing Thr17Ala shows significantly lower ability to inhibit T-cell proliferation and activation than its CTLA4-17Thr counterpart, meaning that a stronger immune response is obtained with the rs231775 GG genotype^17^. The study conducted by Guo, it was showed that the CTLA4 gene +49A>G polymorphism may be associated with a risk of kidney rejection, especially in the Asian population^19^. However, the results obtained by Kim et al., showed no association between this polimorphism and AR^16^. Another study also demonstrated no correlation between CTLA4 and AR^20^. Other reports suggest that the combination of the low AT repeat (82 bp) variant and the homozygous A variant (adenine) in CTLA4 +49A>G polymorphism is associated with a higher estimated glomerular filtration rate (eGFR) 1 year after kidney transplant. People with the AA genotype of CTLA4 +49A>G and a homozygous variant with a low number of AT replicates showed better allograft function up to 10 years after transplant than patients with the +49A>G GG genotype and a homozygous variant of the high number of AT repeats^21^.

Furthermore, TGF-β1 gene polymorphisms have been shown to increase the risk of acute and chronic rejection of a transplanted kidney due to increased expression of this cytokine^21,22^. There are two single nucleotide polymorphisms in the first exon of the TGF-β1 gene which increase the expression of the gene: +869T>C and +915G>C. In vitro studies showed that the genetic polymorphisms of TGF-β1 codon 10 T>C and codon 25 C>G result in increased production of this cytokine. The highest expression was observed in TT homozygous for TGF-β1 codon 10 T>C and GG genotype for TGF-β1 codon 25 C>G^23^. In addition, numerous studies demonstrated an association of the TGF-β1 gene with chronic renal transplant rejection and CsA toxicity^24–26^. Morris-Stiff in the experiments carried out in 2005 recognized TGF-β1 as a fibrogenic cytokine which is involved in fibrosis or chronic rejection of the transplanted kidney^27^. Nikolova et al., showed an increased risk of chronic interstitial fibrosis with tubular atrophy in the carriers of the TT homozygous genotype in codon 10 and homozygotes with a combination of TT codon 10 and GG codon 25. In vivo production of TGF-β1 is stimulated by cyclosporin A, suggesting that this regulation may induce the progression of chronic sclerosis in transplanted organs. However, in most of the available studies, no correlation was found between TGF-β1 polymorphisms and acute transplant rejection^21^. A study conducted by Tinckam et al., showed that the +915G allele has a protective effect on the occurrence of acute transplant rejection. Their analysis concerned recurrent acute rejection 3 months after the transplant^28^. Lacha et al., stated that reduced TGF-β1 expression is a risk factor for rejection in the early postoperative period. Different reports regarding the +869 CC genotype in codon 10, which decreased the TGF-β1 expression and increased the risk of AR in carriers of the G allele (codon 25, + 915GG), except for those who were simultaneously +869TC heterozygous, were also published^29^. In clinical studies, a higher level of TGF-β1 expression was observed in renal transplant biopsy from patients with chronic CsA nephrotoxicity as compared to patients with acute cell rejection or acute tubular necrosis. The primary mechanism of cyclosporin nephrotoxicity is associated with increased expression of the TGF-β1 gene. This cytokine is responsible for causing fibrotic changes in transplanted kidneys, free radical production, as well as increased apoptosis. Recent studies proved that CsA and TAC can stimulate the receptor for transforming growth factor, regardless of the calcineurin pathway, due to increased production of reactive oxygen species and activation of latent TGF-β1, which results in the initiation of the Smad cascade and expression of genes associated with renal fibrotic processes^30–33^. Additionally, various studies indicate that TAC treatment significantly increases the intrarenal expression of TGF-β1 as compared to patients treated with CsA. In addition, TGF-β1 mRNA expression was significantly higher in people diagnosed with nephrotoxicity than in acute rejection^33^. A study conducted among 53 patients treated with CsA and 37 patients treated with TAC after kidney transplant showed that CsA patients with the TC genotype of the TGF-β1 gene codon 10 polymorphism had lower CsA blood concentrations than those with TT and CC genotypes at 1 month. The concentration-dose (C/D) ratio of CsA in the blood in patients with TC genotype was lower than in people with the TT and the CC variants^34^. Additionally, in our study we observed that bilirubin was significantly elevated in CsA than TAC groups (CsA: 0.60 ± 0.28 mg/dL vs TAC 12 h: 0.50 ± 0.29 mg/dL, TAC 24 h: 0.51 ± 0.24 mg/dL). This could be explained by the fact that ciclosporin may cause a dose-dependent increase in serum bilirubin, usually in people with underlying diseases or risk factors. Hence, liver function should be carefully monitored, and a dose reduction may be necessary if disturbances occur.

Moreover, analysis of our results on TAC dose dependence and genetic variants of the TGF-β1 −800G>A polymorphism revealed that patients with the GA genotype required lower doses of the drug as compared to the GG genotype. Comparing the C/D ratio, we showed that in both groups the C/D ratio was higher for patients with the GA genotype. These results indicate a correlation between patients with the A allele and a slightly lower dose of TAC. No relationship was found between individual genotypes and drug dose as far as CsA dose and genetic variants of the TGF-β1 −800G>A polymorphism were concerned.

In conclusion, the results of our study suggest that the TGF-β1 −800G>A polymorphism may influence the dose of TAC, while the CTLA4 +49A>G polymorphism does not correlate with the dose of CsA or TAC. In addition, the evaluation of the clinical parameters (BMI, systolic and diastolic pressure) and the biochemical parameters on the renal profile showed no effect of the CTLA4 and TGF-β1 polymorphisms on the risk of organ rejection because the levels of creatinine, eGFR, and potassium were prognostic of a successful transplantation. Moreover, proteinuria, which falls under the risk of transplant rejection criteria, has not been observed in our patients. In conclusion, there is still a need to look for new genetic factors which increase the risk of acute and chronic rejection of the transplanted organs, as well as to develop an optimal dosing regimen for immunosuppressants based on the patient genetic profile**.**
